# Large vessel vasculopathy in a patient with systemic lupus erythematosus: a case report

**DOI:** 10.1186/s13256-019-2126-4

**Published:** 2019-06-22

**Authors:** Daisuke Waki, Akira Onishi, Akio Morinobu

**Affiliations:** Department of Rheumatology and Clinical Immunology, Kobe University Graduate School of Medicine, 7-5-1 Kusunoki-cho, Chuo-ku, Kobe, 650-0017 Japan

**Keywords:** Systemic lupus erythematosus, Vasculopathy, Vasculitis, Large vessel vasculopathy

## Abstract

**Background:**

Vasculopathy in systemic lupus erythematosus is a rare form of vascular involvement characterized by non-inflammatory vascular injury with the accumulation of immune complexes in the walls of the arteries, resulting in luminal narrowing. While previous reports have demonstrated vasculitis in the large vessels or vasculopathy in the small vessels, vasculopathy in large vessels has not yet been reported.

**Case presentation:**

We present the case of a 43-year-old Japanese woman with peripheral large vessel vasculopathy associated with systemic lupus erythematosus. She presented a 7-year history of progressive headaches and intermittent claudication, although she had no atherosclerotic risk factors. Vascular ultrasonography and enhanced computed tomography showed multiple vascular stenoses and occlusion. The histological findings of her left temporal artery revealed narrowing of the lumen caused by intimal thickening without inflammatory cells and the deposition of immunoglobulin G, complement component 3, and fibrinogen in the wall of the intima. Beraprost and cilostazol improved arterial occlusion without immunosuppressive therapy.

**Conclusions:**

Large vessel vasculopathy should be considered another potential cause of arterial stenoses and occlusion in patients with lupus when they have peripheral arterial disease despite having no atherosclerotic risk factors.

## Background

Vasculopathy in systemic lupus erythematosus (SLE) is a rare form of vascular involvement characterized by arterial stenosis or occlusion, with the accumulation of immunoglobulins and complement in the arterial intima and no inflammatory change [1]. Vasculopathy should be distinguished from vasculitis histopathologically, as vascular lesions in lupus kidney disease can be categorized as non-inflammatory vascular immune complex deposits (vasculopathy), inflammatory vasculitis, thrombotic microangiopathies, and degenerative disorders/arteriosclerosis [1]. While previous studies have reported vasculitis in large vessels or vasculopathy in small vessels of the kidney in SLE, large vessel vasculopathy has not been reported.

Here we present, to the best of our knowledge, the first case of peripheral large vessel vasculopathy associated with SLE. The possibility of large vessel vasculopathy should be considered in a patient with lupus when the patient has peripheral arterial disease, despite having no atherosclerotic risk factors.

## Case presentation

**Fig. 1:**
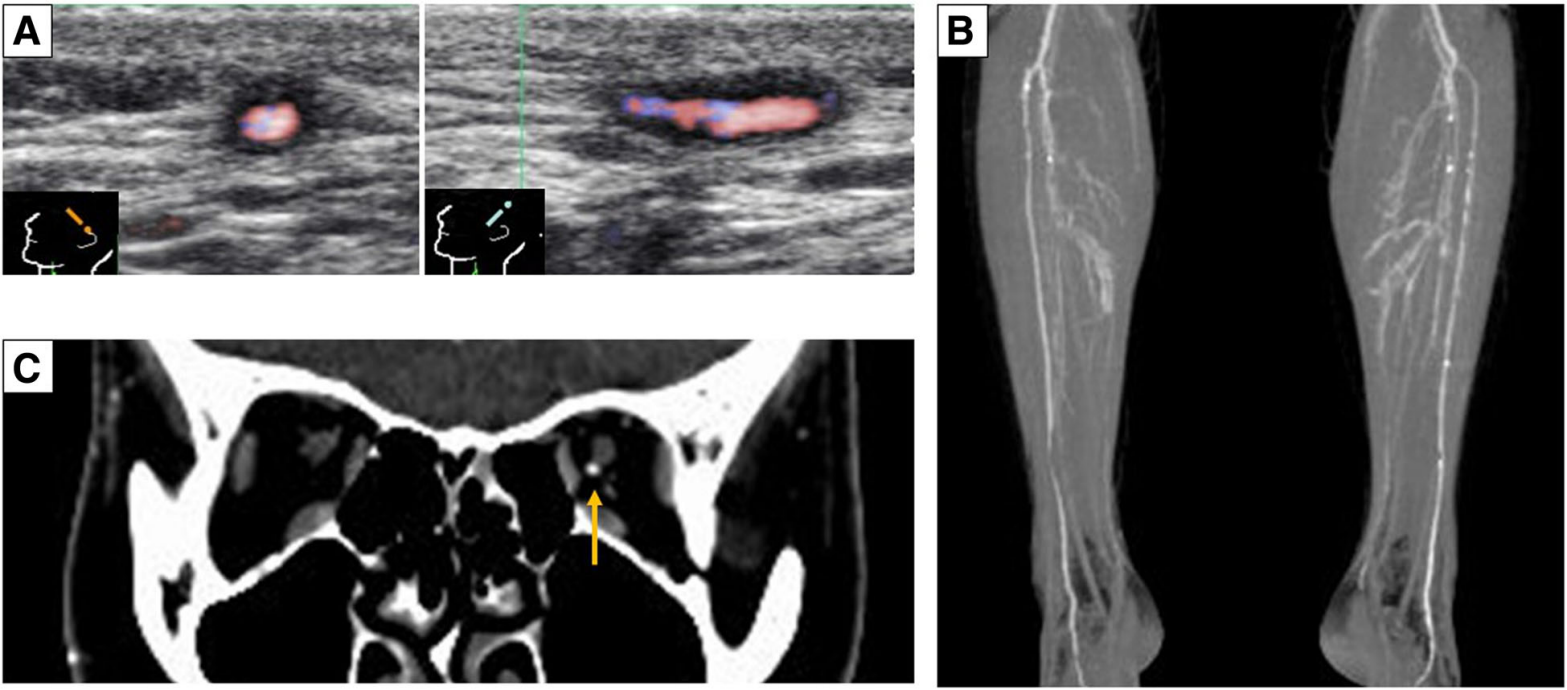
Multiple vascular stenoses and occlusion. **a** Vascular ultrasonography revealed concentric hypoechoic mural thickening (the halo sign) in the left temporal artery. **b** Reconstruction image of an enhanced computed tomography scan showed bilateral occlusion of the posterior tibial arteries and the peroneal arteries. **c** Enhanced computed tomography showed occlusion of the right ophthalmic artery while the left ophthalmic artery was normally detected (*yellow arrow*)

A 43-year-old Japanese woman presented to our hospital with a 7-year history of progressive headaches and intermittent claudication. She was diagnosed as having SLE according to the 1997 American College of Rheumatology revised classification criteria (oral ulcers, discoid rashes, positive anti-nuclear antibody, leukopenia, and lymphopenia) [2] and histological findings of the skin (interface dermatitis and positive lupus band test) 11 years previously, all of which improved with low-dose prednisolone. She had no atherosclerotic risk factors such as cigarette smoking, dyslipidemia, diabetes mellitus, or hypertension. She drank alcohol only on social occasions and had no significant other past medical, social, environmental, obstetrical, gynecological, or employment history. Her mother had autoimmune hepatitis and died at the age of 60 of subarachnoid hemorrhage. On hospitalization, her temperature was 36.0 °C, her blood pressure was 124/82 mmHg, and her pulse was 78 per minute, regular and of normal tension. A physical examination revealed the absence of pulsation in the bilateral posterior tibial arteries and the left dorsalis pedis artery. She had no adenopathy, aphthous ulcers, or rash. Her heart sounds were clear and regular without audible murmurs and her lungs were clear. Her abdomen was flat and soft, and bowel sounds were normal without murmurs. A neurological examination showed that her cranial nerves were intact, her muscle strength was normal, her deep tendon reflexes were symmetrical without Babinski sign, and no sensory abnormalities were noted. Laboratory data revealed mild leukopenia (3300/μL) and slightly elevated erythrocyte sedimentation rate (24 mm/hour). C-reactive protein level, complement level, and urine sediment were normal. Anti-double-stranded deoxyribonucleic acid (dsDNA), anti-Smith, anti-cardiolipin, anti-neutrophil cytoplasmic antibodies, and lupus anticoagulant were all negative. Vascular ultrasonography revealed occlusion of her bilateral posterior tibial arteries, bilateral peroneal arteries, and left anterior tibial artery with collateral circulation and wall thickening of her right radial artery. Luminal narrowing in her right temporal artery and concentric hypoechoic mural thickening (the halo sign) in her left temporal artery were detected, although no abnormalities in her carotid arteries were found (Fig. 1A).

**Fig. 2:**
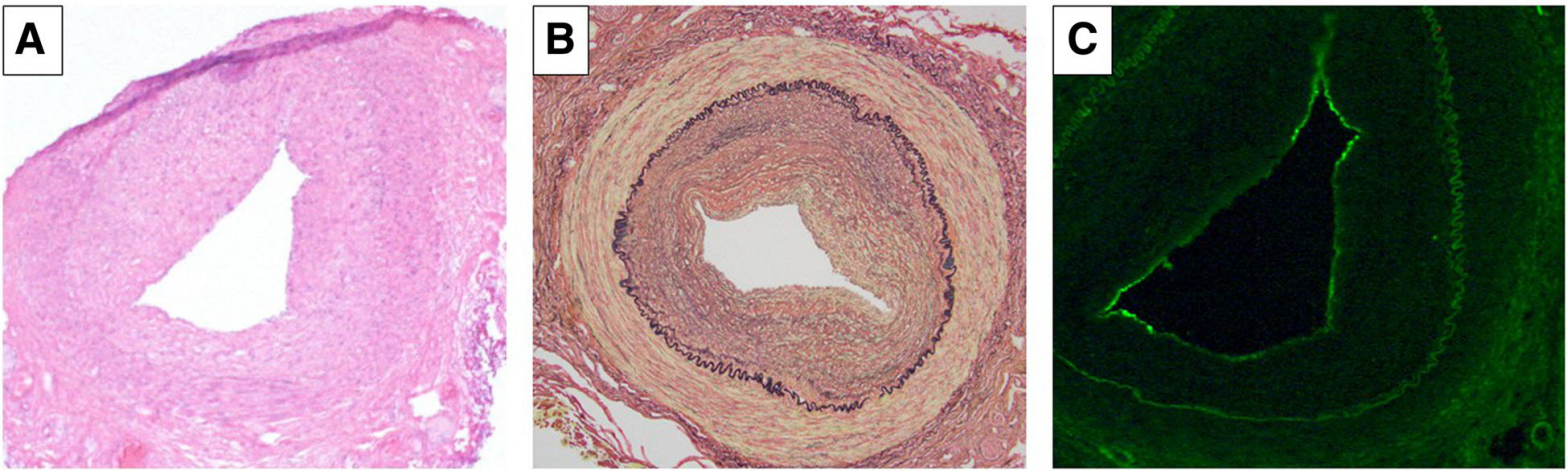
Histological findings of the left temporal artery. **a** Hematoxylin and eosin staining revealed a lumen narrowed by intimal thickening, without inflammatory cells. There was no evidence of atherosclerotic findings (lipid accumulations and fibrous caps), Monckeberg’s calcification, or active temporal arteritis. **b** Elastica-van Gieson staining did not show fragmentation and loss of elastic fibers in the internal elastic lamina. **c** The wall of the intima was stained with C3 and evaluated by immunofluorescence

Enhanced computed tomography showed vascular occlusion of bilateral lower limbs and her right ophthalmic artery (Fig. 1B, C). Enhanced magnetic resonance imaging and positron emission tomography revealed no inflammation in the wall of the large vessels. The histological findings of the left temporal artery revealed narrowing of the lumen caused by intimal thickening without inflammatory cells, and the deposition of immunoglobulin G (IgG), complement component 3 (C3), and fibrinogen in the wall of the intima (Fig. 2). Atherosclerotic changes, such as fibrous plaque, foam cells, or Monckeberg’s calcification, were not found. Active or healed arteritis, including giant cells within the intima, fragmentation, loss of internal elastic lamina, or neovascularization of the media, was not detected.

We made a diagnosis of large vessel vasculopathy associated with SLE based on the histopathological findings. Vascular involvements were only observed in the peripheral large vessel according to the Chapel Hill Consensus Conference nomenclature system [3]. We initiated 120 μg/day of beraprost sodium and 200 mg/day of cilostazol therapy orally. At 6 months after discharge, re-examination by ultrasonography revealed recanalization in our patient’s left posterior tibial artery.

## Discussion and conclusions

To the best of our knowledge, this is the first report of peripheral large vessel vasculopathy associated with SLE and demonstrates two important points.

First, vasculopathy, as well as vasculitis, may occur in the large vessel. Although several reports showed large vessel involvements in SLE, all of them demonstrated vasculitis or thrombosis of large vessel [4–8]. Takagi *et al.* reported a case of SLE with non-dissecting aneurysm that was successfully resected, replaced with a tube graft, and proven to be active aortitis [6]. In this patient, focal calcification and atheroma were found within the thickened intima, which contained activated T and B lymphocytes in a perivascular lesion. However, some previous publications might include cases with large vessel vasculopathy especially when they were diagnosed only with angiography because vasculopathy is often confused with vasculitis. In contrast, the present case showed only a thickened intima with immunoglobulins and complement deposition, without any inflammatory lymphocyte infiltration or atherosclerotic change, which are typical features of vasculopathy.

Second, the present case had vasculopathy in the large vessel, not the small or medium vessel. Lupus vasculopathy is commonly found in the kidney. Pathological findings in renal lupus vasculopathy are mainly present in small vessels, such as the pre-glomerular arterioles and small arteries [1]. In addition, although cases of extra-renal vasculopathy in lupus were rarely reported, all were shown to involve the small vessels [9–11]. In contrast, the present case was demonstrated to have peripheral large vessel vasculopathy based on temporal artery biopsy.

Several limitations should be noted. First, a previous use of prednisolone might modulate pathological findings. Differential diagnoses of large vessel vasculopathy include atherosclerosis and healed vasculitis. Although accelerated atherosclerosis is a common complication in SLE [12], the pathological findings of the present case did not show atherosclerotic changes such as fatty streak or atheroma [13, 14]. In addition, healed temporal arteritis was less likely to occur in this case as there were no neovascularizations of the media or loss of the internal elastic lamina [15]. Second, we could not speculate regarding the incidence or prevalence of vasculopathy based on the nature of the case reports. Some peripheral large vessel vasculopathy may, however, remain unrecognized among patients with SLE with peripheral arterial diseases. Thus, further studies are needed to determine the prevalence of vasculopathy among patients with SLE with peripheral arterial disease.

The present case was treated without immunosuppressive therapy. Although previous cases of lupus vasculopathy were mainly treated with immunosuppressive therapy because of coincidental active lupus involvement or its hypothesized mechanism, it has not been established whether conventional immunosuppressive agents are effective [1, 10]. Sugimoto *et al.* reported a case of renal lupus vasculopathy without active glomerular lesions successfully treated without immunosuppressive therapy [16]. The patient, who had a previous medical history of central nervous system lupus, developed acute renal infarction and multiple arterial stenoses in the interlobular arteries. Because no active inflammatory changes were found during renal biopsy, they did not initiate immunosuppressive therapy, which resulted in the improvement of renal dysfunction. The present case also experienced improvement of arterial occlusion without immunosuppressive therapy. Further reports should be accumulated to determine whether immunosuppressive agents are needed to treat lupus vasculopathy without active inflammation.

In conclusion, peripheral large vessel vasculopathy may occur in patients with SLE. Physicians should consider the possibility of large vessel vasculopathy in a patient with lupus when the patient has peripheral arterial disease, despite having no atherosclerotic risk factors.

## Data Availability

Because this is a case report of a single patient, there are no available raw data, this is to protect her privacy and respect confidentiality. The original reports, laboratory studies, imaging studies, and out-patient clinic records are retained as per normal procedure within the medical records of our institution.

## Abbreviation

SLE: Systemic lupus erythematosus
